# 

**Published:** 1900-11

**Authors:** 


**This Journal and Atlanta Semi-weekly Journal one year for $1.00 cash in advance. No checks. Strictly to new subscribers.**

### *OTHER BOOKS RECEIVED.*

---

A Treatise on Fractures and Dislocations. For Practitioners and Students. By Lewis A. Stimson, B.A., M.D., Professor of Surgery in Cornell University Medical College, New York. New (3d) edition. In one 8vo volume of 842 pages, with 336 engravings and 32 full-page plates. Philadelphia and New York: Lea

Brothers & Co. 1900. Price, cloth, $5.00 net; leather, $6.00 net.

A Text-Book of the Practice of Medicine. By James M. Anders, M.D., Ph.D., LL.D., Professor of the Practice of Medicine and of Clinical Medicine in the Medico-Chirurgical College, Philadelphia; Attending Physician to the Medico-Chirurgical and Samaritan Hospitals, Philadelphia. Octavo, pp. 1263. Illustrated. Fourth edition, thoroughly revised. Philadelphia and London: W. B. Saunders & Co. 1900. Price, cloth, $5.00 net; sheep and half Morocco, $6.50.

Modern Surgery. General and Operative. By John Chalmers DaCosta, M.D., Professor of the Principles of Surgery and of Clinical Surgery, Jefferson Medical College, Philadelphia; Surgeon to the Philadelphia Hospital and to St. Joseph's Hospital, Philadelphia. Octavo, pp. 1117. With 493 illustrations. Third edition, revised and enlarged. Philadelphia and London: W. B. Saunders & Co. 1900. Price, cloth, $5.50 net; half Morocco, $6.00.

Modern Medicine. By Julius L. Salinger, M.D., Demonstrator of Clinical Medicine, Jefferson Medical College; Hematologist to the Jefferson Medical College Hospital; Pathologist to the Lying-in Charity Hospital, Philadelphia; Assistant Pathologist to the Philadelphia Hospital. Octavo, pp. 801. Illustrated. Philadelphia and London: W. B. Saunders & Co. 1900. Price, cloth, $4.00 net.

Twentieth Century Practice. An International Encyclopedia of Modern Medical Science. By Leading Authorities of Europe and America. Edited by Thomas L. Stedman, M.D., New York City. In twenty volumes. Volume XX. "Tuberculosis, Yellow Fever and Miscellaneous. General Index." New York: William Wood & Company. 1900.

A Text-Book of the Diseases of Women. By Henry J. Garrigues, A.M., M.D., Gynecologist to St. Mark's Hospital, New York City. Octavo volume of 756 pages, with 367 illustrations. Third edition, thoroughly revised. Philadelphia and London: W. B.

Saunders & Co. 1900. Price, cloth, $4.00 net; sheep or half Morocco, $5.50 net.

Diet Lists and Sick-Room Dietary. By Jerome B. Thomas, M.D., Visiting Physician to the Home for Friendless Women and Children and to the Newsboys' Home; Assistant Bacteriologist, Brooklyn Health Department. Philadelphia: W. B. Saunders & Co. 1900. Price, cloth, $1.50.

Taylor on Genito-Urinary and Venereal Diseases and Syphilis. The Pathology and Treatment of Genito-Urinary and Venereal Diseases and Syphilis. By Robert W. Taylor, A.M., M.D., Clinical Professor of Venereal Diseases in the College of Physicians and Surgeons, New York. New (2d) edition. Octavo volume of 720 pages, with 135 engravings and 27 full-page plates in colors and monotone. Philadelphia and New York: Lea Brothers & Co. 1900. Cloth, $5.00 net; leather, $6.00.

Les Maladies Qu'on Soigne A. Berck. Abcès froids, adénites, ostéites, Tumeurs Blanches, Coxalgie, Mal de Pott, Scoliose, Luxation Congénitale de la Hanche, Pied Bot, etc. Par F. Calot, Chirurgien en chef de l'Hopital Rothschild, etc., etc. Pages, 433. Paris: G. Masson, Editeur, Librairie de L'Academie de Médecine, 120 Boulevard Saint Germain. 1900.

A Text-Book Upon the Pathogenic Bacteria. For Students of Medicine and Physicians. By Joseph McFarland, M.D., Professor of Pathology in the Medico-Chirurgical College, Philadelphia. Octavo, 621 pages, illustrated. Third edition, revised and enlarged. Philadelphia and London: W. B. Saunders & Co. 1900. Price, cloth, $3.25 net.

Pathology and Morbid Anatomy. By T. Henry Green, M.D., F.R.C.P., Physician and Special Lecturer on Clinical Medicine at Charing Cross Hospital, etc. New (9th) American from ninth English edition. Revised and enlarged by H. Montague Murray, M.D., F.R.C.P., Lecturer on Pathology and Morbid Anatomy at Charing Cross Hospital. Revised for America by Walton Martin, Ph.B., M.D., of the College of Physicians and Surgeons, New York City. Octavo volume of 578 pages, with 4 colored plates and 339 engravings. Philadelphia and New York: Lea Brothers & Co. Price, cloth, $3.25 net.

The American Illustrated Medical Dictionary. A New and Complete Dictionary of the Terms Used in Medicine, Surgery, Dentistry, Pharmacy, Chemistry and the Kindred Branches. With their Pronunciation, Derivation, and Definition. Including much Collateral Information of an Encyclopedic Character. By W. A. Newman Dorland, A.M., M.D., Assistant Obstetrician to the University of Pennsylvania Hospital; Editor of the American Pocket Medical Dictionary. With numerous illustrations and 24 colored plates. Philadelphia and London: W. B. Saunders & Co. 1900. Price, $4.50, plain; $5.00, index.

A Text-Book of Pathology. By Alfred Stengel, M.D., Professor of Clinical Medicine in the University of Pennsylvania; Physician to the Philadelphia Hospital; Physician to the Children's Hospital, Philadelphia, etc. Octavo, pp. 873. With 372 illustrations. Third edition, revised. Philadelphia and London: W. B. Saunders & Co. 1900. Price, cloth, $5.00 net; half-Morocco, $6.00 net.

Saunders's Question Compends. Essentials of Histology. By Louis Leroy, B.S., M.D., Professor of Histology and Pathology, in Vanderbilt University, Medical and Dental Departments; City Bacteriologist to Nashville, Tenn.; Bacteriologist to the State of Tennessee. Arranged with questions following each chapter. Duodecimo, pp. 231, 72 illustrations. Philadelphia: W. B. Saunders & Co. 1900. Price, $1.00 net.

Musser's Diagnosis. New (4th) edition. A Practical Treatise on Medical Diagnosis. For the Use of Students and Practitioners. By John H. Musser, M.D., Professor of Clinical Medicine, University of Pennsylvania, Philadelphia. New (4th) edition, thoroughly revised. In one octavo volume of 1104 pages, with 250 engravings and 49 full-page colored plates. Cloth $6.00 net; half-Morocco, $7.50 net. Lea Brothers & Co., Publishers, Philadelphia and New York. October, 1900.

***NOTICE.—This JOURNAL and THE INTERNATIONAL JOURNAL OF SURGERY one year for $1.00 cash, to new subscribers. Out-of-town checks not accepted unless 10c. is added for cost of collection. Strictly to new subscribers.***

***NOTICE.—This JOURNAL and THE INTERNATIONAL JOURNAL OF SURGERY one year for $1.00 cash, to new subscribers. No out-of-town checks accepted unless 10c. is added for cost of cashing. Strictly to new subscribers.***

